# In the Ant's Garden, Fungal Parasites Are Closely Matched to Their Fungal Hosts

**DOI:** 10.1371/journal.pbio.0040259

**Published:** 2006-07-11

**Authors:** Richard Robinson

Like all gardeners, fungus-growing ants risk losing their crop to pests. In particular, the cultivated fungi are parasitized by other fungi—of the species *Escovopsis*—which invade the fungal garden and consume the crop within. In virtually all cases, a single species of ant grows only a narrow range of genetically similar fungal cultivars, and these, in most cases, are parasitized by a few or only one type of *Escovopsis*.

While the ants do their best to protect their crop, it is important for the fungus itself to ward off the depredations of *Escovopsis*. Exactly how they do this is unknown. In a new study, Nicole Gerardo and colleagues demonstrate that different fungal cultivars have different abilities to ward off potential parasites; that successful parasites can best overcome the defenses of that cultivar they typically infect; and that host-switching by *Escovopsis* is likely inhibited by the genetically based specificity of these relationships.

The authors first showed that growth of the various *Escovopsis* types toward their preferred fungal cultivars is driven by a signal from the target. Yellow-spored *Escovopsis* grew faster toward its preferred host (cultivar A) than toward no target, or toward cultivar B, which it does not feed on. Brown-spored *Escovopsis*, which feeds on both A and B, grew faster toward either than toward no target. Neither the yellow nor the brown types did well against cultivar C, which is only distantly related to their normal hosts. In many cases, cultivar C kept these predators at bay for up to several months.

In some trials, however, C did not repel the yellow type and, in fact, in some trials, the yellow fungus grew faster toward C. This suggests genetic variability in the two fungal groups, which the authors confirmed. They showed that genetically similar *Escovopsis* strains were likely to be inhibited by the same C cultivar, and vice versa—genetically similar C cultivars were likely to inhibit a specific *Escovopsis* strain.

Over time, such close relationships, and the difficulty of overcoming non-host defenses, may inhibit host switching on the part of *Escovopsis*, and lead to synchronous speciation of the fungal hosts and parasites. On the other hand, the genetic variation that allowed some yellow *Escovopsis* to attack the C cultivars suggests that host switching remains a possibility among these fungi.

These results suggest that the ability of a specific *Escovopsis* type to parasitize a specific fungal cultivar is aided both by the chemical cues that attract the parasite and the close match between the host's defensive weaknesses and the parasite's strengths. The rapid growth this allows may help the parasite to stay ahead of the ant's anti-fungal defensive mechanisms (like any good gardener, the ant farmers weed out the species they don't want in their garden). More broadly, these results help clarify the variety of mechanisms involved in host–parasite relationships, which exist across all the domains of life.

## 

**Figure:**
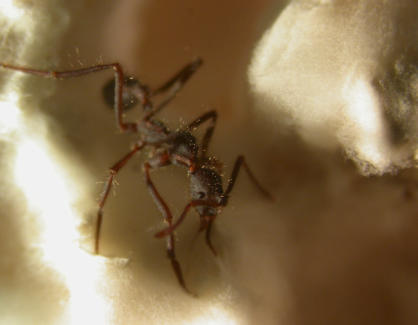
Different pathogens of the Escovopsis fungus show highly specialized preferences for different strains of fungi cultivated by ants.

